# Using a whole-body ^31^P birdcage transmit coil and 16-element receive array for human cardiac metabolic imaging at 7T

**DOI:** 10.1371/journal.pone.0187153

**Published:** 2017-10-26

**Authors:** Ladislav Valkovič, Iulius Dragonu, Salam Almujayyaz, Alex Batzakis, Liam A. J. Young, Lucian A. B. Purvis, William T. Clarke, Tobias Wichmann, Titus Lanz, Stefan Neubauer, Matthew D. Robson, Dennis W. J. Klomp, Christopher T. Rodgers

**Affiliations:** 1 Oxford Centre for Clinical Magnetic Resonance Research (OCMR), BHF Centre of Research Excellence, University of Oxford, Oxford, United Kingdom; 2 Department of Imaging Methods, Institute of Measurement Science, Slovak Academy of Sciences, Bratislava, Slovakia; 3 Siemens Healthcare Limited, Frimley, United Kingdom; 4 MR Coils BV, Zaltbommel, Netherlands; 5 Rapid Biomedical GmbH, Rimpar, Germany; 6 Department of Radiology, University Medical Center Utrecht, Utrecht, Netherlands; Linköping University, SWEDEN

## Abstract

**Purpose:**

Cardiac phosphorus magnetic resonance spectroscopy (^31^P-MRS) provides unique insight into the mechanisms of heart failure. Yet, clinical applications have been hindered by the restricted sensitivity of the surface radiofrequency-coils normally used. These permit the analysis of spectra only from the interventricular septum, or large volumes of myocardium, which may not be meaningful in focal disease. Löring et al. recently presented a prototype whole-body (52 cm diameter) transmit/receive birdcage coil for ^31^P at 7T. We now present a new, easily-removable, whole-body ^31^P transmit radiofrequency-coil built into a patient-bed extension combined with a 16-element receive array for cardiac ^31^P-MRS.

**Materials and methods:**

A fully-removable (55 cm diameter) birdcage transmit coil was combined with a 16-element receive array on a Magnetom 7T scanner (Siemens, Germany). Electro-magnetic field simulations and phantom tests of the setup were performed. In vivo maps of B_1_^+^, metabolite signals, and saturation-band efficiency were acquired across the torsos of eight volunteers.

**Results:**

The combined (volume-transmit, local receive array) setup increased signal-to-noise ratio 2.6-fold 10 cm below the array (depth of the interventricular septum) compared to using the birdcage coil in transceiver mode. The simulated coefficient of variation for B_1_^+^ of the whole-body coil across the heart was 46.7% (surface coil 129.0%); and the in vivo measured value was 38.4%. Metabolite images of 2,3-diphosphoglycerate clearly resolved the ventricular blood pools, and muscle tissue was visible in phosphocreatine (PCr) maps. Amplitude-modulated saturation bands achieved 71±4% suppression of phosphocreatine PCr in chest-wall muscles. Subjects reported they were comfortable.

**Conclusion:**

This easy-to-assemble, volume-transmit, local receive array coil combination significantly improves the homogeneity and field-of-view for metabolic imaging of the human heart at 7T.

## Introduction

Phosphorus magnetic resonance spectroscopy (^31^P-MRS) plays an important role in the assessment of tissue metabolism, through measurement of high-energy metabolites, such as phosphocreatine (PCr) and adenosine triphosphate (ATP), in vivo [1, 2]. The PCr/ATP ratio is of particular interest in cardiovascular medicine, serving as a valuable biomarker that changes in most major heart diseases [3–5], and which even predicts mortality in patients with dilated cardiomyopathy [3]. Impaired cardiac PCr/ATP ratios also occur in systemic diseases, such as type-II diabetes [6] and obesity [7].

Yet ^31^P-MRS is still applied in clinical medicine, principally because of its comparatively long scan times, signal-to-noise ratios that are too low for single-subject comparisons, and the challenges of recording regionally-resolved spectra across the heart.

Moving to ultra-high field (i.e. 7T) from clinical MR systems (i.e. ≤3T) leads to a 2.5-fold increase in SNR [8, 9] and increased spectral resolution [10, 11]. The use of dedicated receive-arrays further improves the SNR and extends coverage to more of the heart at 7T [5]. However, so far receive array coils for cardiac ^31^P-MRS have used surface coils for transmission. Surface coils’ radiofrequency (RF) transmit field strength (B_1_^+^) inherently drops-off rapidly with increasing distance from the coil to the volume of interest [12, 13]. This makes spatially-resolved ^31^P-MRS imaging (^31^P-MRSI) across the heart challenging. Even with custom-built surface transmit coils, and optimised adiabatic B_1_^+^-insensitive excitation pulses, regionally-resolved measurement across the whole heart in clinically feasible times remains elusive [14].

Recently, Löring et al inserted a whole-body-sized (52 cm in diameter) ^31^P birdcage RF-coil into the bore of a Philips 7T MR system (Philips Healthcare, Best, Netherlands). This showed relatively uniform spectral profiles for ^31^P-MRSI examinations with a rectangular RF pulse in a cylindrical phantom and in a human subject [15]. However, their coil had to be inserted into the magnet bore after complete removal of the original patient bed. They also used the RF screen inside the patient tube decreasing the patient space significantly and affecting subject comfort and study inclusion criteria. An alternative design, not requiring complex preparation, and allowing fast installation/removal would be preferred. Furthermore, the previously described proof-of-concept coil operated only in transceiver mode, which led to low resolution MRSI matrixes in order to compensate for the inherently low SNR.

In this study, we report initial results of a collaborative project to design, build and test a new, easily-removable, high-pass birdcage, whole-body (55cm-diameter) ^31^P transmit RF-coil for use on a Magnetom 7T MR scanner (Siemens Healthcare, Erlangen, Germany), integrated into an extension of the scanner’s motorized patient bed; we use it in conjunction with a 16-element anterior receive array (Rapid Biomedical, Rimpar, Germany) for cardiac ^31^P-MRSI at 7T.

## Materials and methods

**Fig 1:**
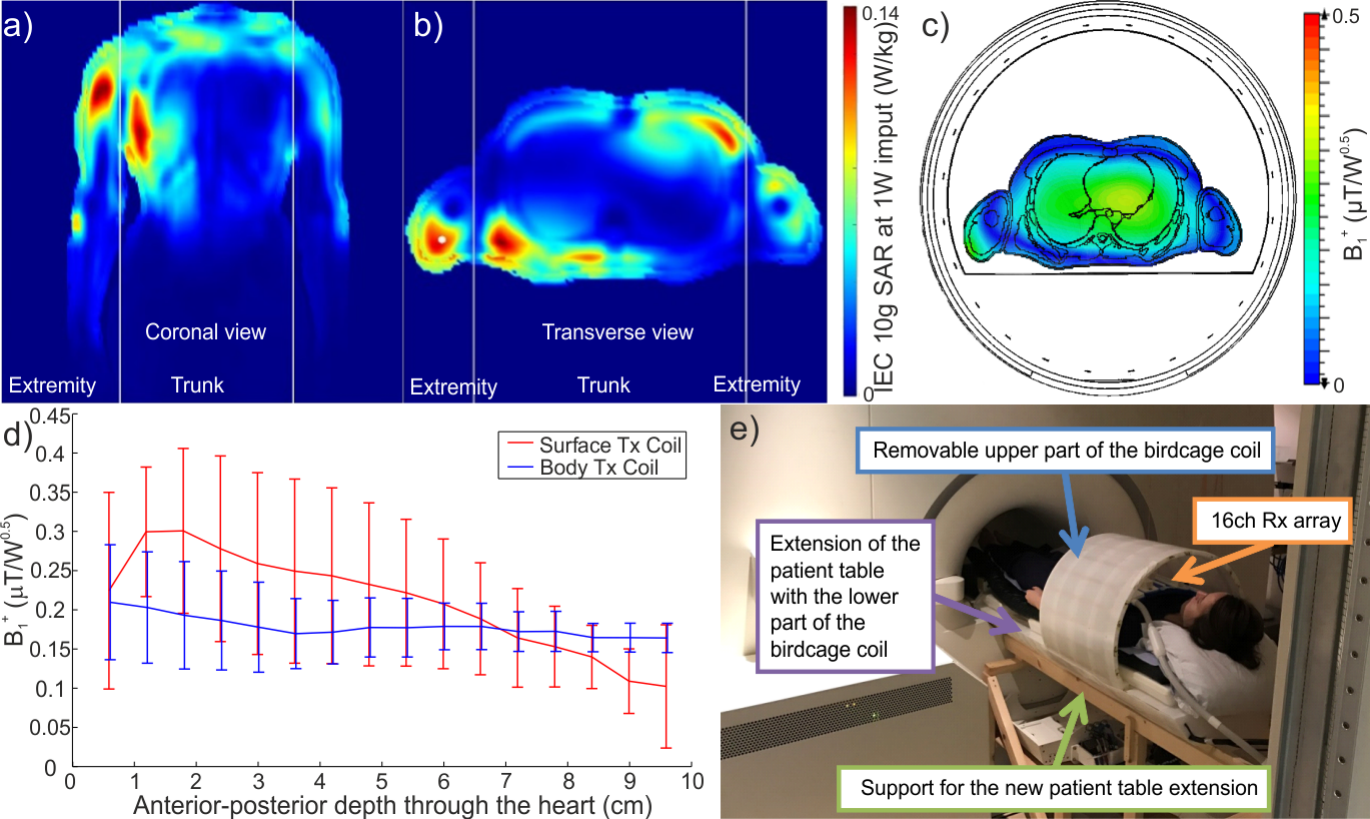
Design and simulations of the new whole-body RF-coil. (a-d) Electromagnetic field modelling results for the designed whole-body coil. (a) Coronal view and (b) transversal view of the 10g local SAR maps showing hotspots in the chest wall and shoulders. (c) Predicted B_1_^+^ efficiency map. Note the uniformity of excitation across the heart and liver. (d) Direct comparison of B_1_^+^ variability across the heart between surface transmit (Tx) coil and the whole-body Tx coil. (e) A photograph of the new apparatus installed on our 7T MRI scanner. The extension of the patient table with the integrated whole-body coil rests on a custom-build support frame and connects to the existing motorized patient bed from the service end of the scanner.

The underlying design of our whole-body coil was similar to that previously reported [15]. However, to allow the desired easily removable setup, the design of the whole-body coil was adjusted by MR Coils (MR Coils BV, Zaltbommel, Netherlands) so that the lower rungs of the birdcage were integrated into an extension of the scanner’s motorised patient bed. This is driven onto custom-built support rails at the service-end of the magnet for subject access (Fig 1E). As the coil had to be inserted without removing the patient tube, while maximizing space for the subject, the existing RF shield integrated in the scanner’s gradient coil was used as the RF shield for the body coil. Therefore, only the rings and rods of the birdcage had to be shifted inside the bore of 58 cm, leading to a setup with inner coil diameter of 55 cm. The upper part of the birdcage coil was made detachable to ease patient positioning. The coil was tuned to 120.3 MHz, the frequency for ^31^P-MR on a Magnetom 7T MR scanner. The standard Siemens 8-kW RF power amplifier was used at this stage.

Simulations of electro-magnetic fields were performed out using CST Studio Suite 2016 (CST Computer Simulation Technology AG, Darmstadt, Germany). A whole-body coil matching the proposed design, i.e. a 40-cm long, 20-rung birdcage coil with a 55 cm diameter, was simulated inside an RF shield matching the diameter and length of the shield of the scanner. Two voxel models, “Gustav” and “Laura”, were simulated in order to identify the 10g local, global head and global body specific absorption rate (SAR) values for 1W input power. In order to get the worst case for the 10g local SAR values, one arm of the voxel model was placed in direct contact with the inner lining of the transmit coil. A rectangular (26×28 cm^2^) surface transmit coil, previously used in our lab for ^31^P excitation at 7T [5], was also simulated.

The coil performance was first tested in a two compartment phantom consisting of a 18 L container (outer dimensions 46×24×17 cm^3^), filled with NaCl_(aq)_ (73 mM), and a 2cm cube, filled with KH_2_PO_4(aq)_ (1.8 M), set at a 10cm depth. A series of fully relaxed (TR = 10 s), non-localized, single average ^31^P-MRS spectra were acquired increasing the peak voltage of a 10 ms rectangular pulse from 25 V to 400 V in 25 V steps. The SNR and peak B_1_^+^ were compared between a quadrature surface ^31^P RF-coil in transceiver mode (two 15 cm diameter loops, with overlap decoupling, as described in [14]), the new whole-body (55-cm diameter) birdcage coil in transceiver mode; and the combination of the whole-body coil for transmission and a 16-element receive array, consisting of 4×4 matrix of overlapping 8×5.5 cm^2^ flexible receive loops [16], (Rapid Biomedical) for reception. Additional tests were performed to check the compatibility of the whole-body coil with the 16-channel receive array, e.g., scattering parameters measurements, and a heating test using fibre-optic temperature probes (Neoptix Inc, Quebec, Canada) and an infrared camera (FLIR Systems Inc, Wilsonville, Oregon). Ultimately, scanning human subjects with the receive array inside the whole-body transmit coil was found to be safe.

Eight healthy volunteers (one female, mean age 28 ± 5 years, ages ranging from 21 to 35 years) were approached between October 2016 and January 2017 and all were consecutively recruited for our three in vivo experiments. A written informed consent was obtained in compliance with ethical and legal requirements. Oxford Central University Research Ethics Committee provided approval for this technical development work. No further demographic characteristics were recorded for the recruited volunteers. The individual whose photograph is shown in Fig 1 has given written informed consent (as outlined in PLOS consent form) to publish this photograph. All subjects were positioned supine inside the whole-body coil and CINE ^1^H FLASH images were acquired using a single ^1^H, transmit/receive, fractionated dipole antenna RF-coil (MR Coils) positioned over the heart. The ^1^H RF-coil was then replaced with the 16-channel receive array for the ^31^P experiments. The first experiment recorded high-quality 3D-resolved spectra using our established cardiac ^31^P-MRS protocol. Specifically: acquisition-weighted 3D-UTE-CSI [17] spectra were acquired over a 16×16×8 matrix covering a 500×500×400 mm^3^ field-of-view (FOV) in three volunteers, using a 1 ms long amplitude-modulated excitation pulse [8]. Eight averages using TR = 1 s were acquired in 23 minutes 52 seconds.

The second experiment recorded metabolite maps and tested the performance of amplitude-modulated RF “saturation bands”. Four volunteers were examined in the second experiment using two acquisition-weighted, transverse 2D-UTE-CSI experiments with a 24×24 matrix over 500×500 mm^3^ FOV, and 60 mm slice thickness. Slice selective 2.5 ms long sinc pulses were used for excitation. Relatively short TR = 300 ms was used allowing for 32 averages within 19 minutes 26 seconds. Two amplitude-modulated saturation bands (10 ms duration) were used to suppress signal from chest muscles in one of the acquisitions.

The third (and final) experiment recorded B_1_^+^ field maps to quantify the transmit B_1_^+^ homogeneity of the new whole-body RF-coil in vivo. Four subjects underwent B_1_^+^ field mapping using a Bloch-Siegert sequence [13] with similar 2D resolution and excitation as in the second experiment. The Fermi pulse (8 ms duration), placed at ±2 kHz from PCr, required a minimum TR = 400 ms. The number of averages was 32 or 48, leading to a scan time of 26 minutes or 38 minutes for each of the ±2 kHz Fermi pulse frequency offsets.

Signals from individual receive elements were combined using whitened singular value decomposition [18], and the combined spectra were fitted using a Matlab (MathWorks, Natick, MA) implementation of the AMARES time domain fitting routine [19]. The B_1_^+^ field maps were calculated in all voxels with sufficient SNR, as defined in [13].

## Results

The results of our simulations are depicted in Fig 1A–1D. The simulated local 10 g SAR efficiency, global SAR efficiency and global head SAR efficiency of the designed coil were 3.4, 0.28 and 0.25 W/kg/μT^2^, respectively. Our simulated worst-case local 10 g SAR in the body was 0.145 W/kg and global body SAR was 0.013 W/kg. The mean simulated B_1_^+^ for 1 W delivered power was 0.160 ± 0.075 μT for the heart and 0.122 ± 0.068 μT for liver (mean ± standard deviation for voxels of tissue type “heart” or “liver” in the 3D results). The coefficients of variation (CV), i.e. the standard deviation divided by the mean, for simulated B_1_^+^ across the “heart” type voxels were 46.7% for the new birdcage coil (55cm-diameter) and 129.0% for the rectangular surface coil.

The results of the phantom SNR and peak B_1_^+^ comparison between the coils are given in Table 1. The SNR of the combined whole-body coil transmit and 16-channel receive was 2.6 times higher in comparison to the whole-body coil in transceiver mode. The peak B_1_^+^ of the combined whole-body transmit and local receivers at a 10 cm depth below the coil was 3.5 times lower compared to the quadrature surface coil, however, the SNR achieved was comparable.

**Table 1:** Comparison of the SNR and peak B1+ between three combinations of RF transmit and receive coils in a phantom placed 10 cm away from the surface RF-coil.

	Quadrature surface transceiver coil	Whole-body transceiver	Whole-body transmit coil with receive array
**Max. signal**	102.48 (2.30)	44.57	126.65 (2.84)
**STD Noise**	3.39 (0.91)	3.73	4.08 (1.09)
**SNR**	30.23 (2.53)	11.95	31.04 (2.60)
**peak B_1_^+^ [μT]**	16.83 (3.00)	5.61	4.81 (0.86)

The values in parentheses represent the ratio of each value to that for the whole-body coil in transceiver mode

**Fig 2:**
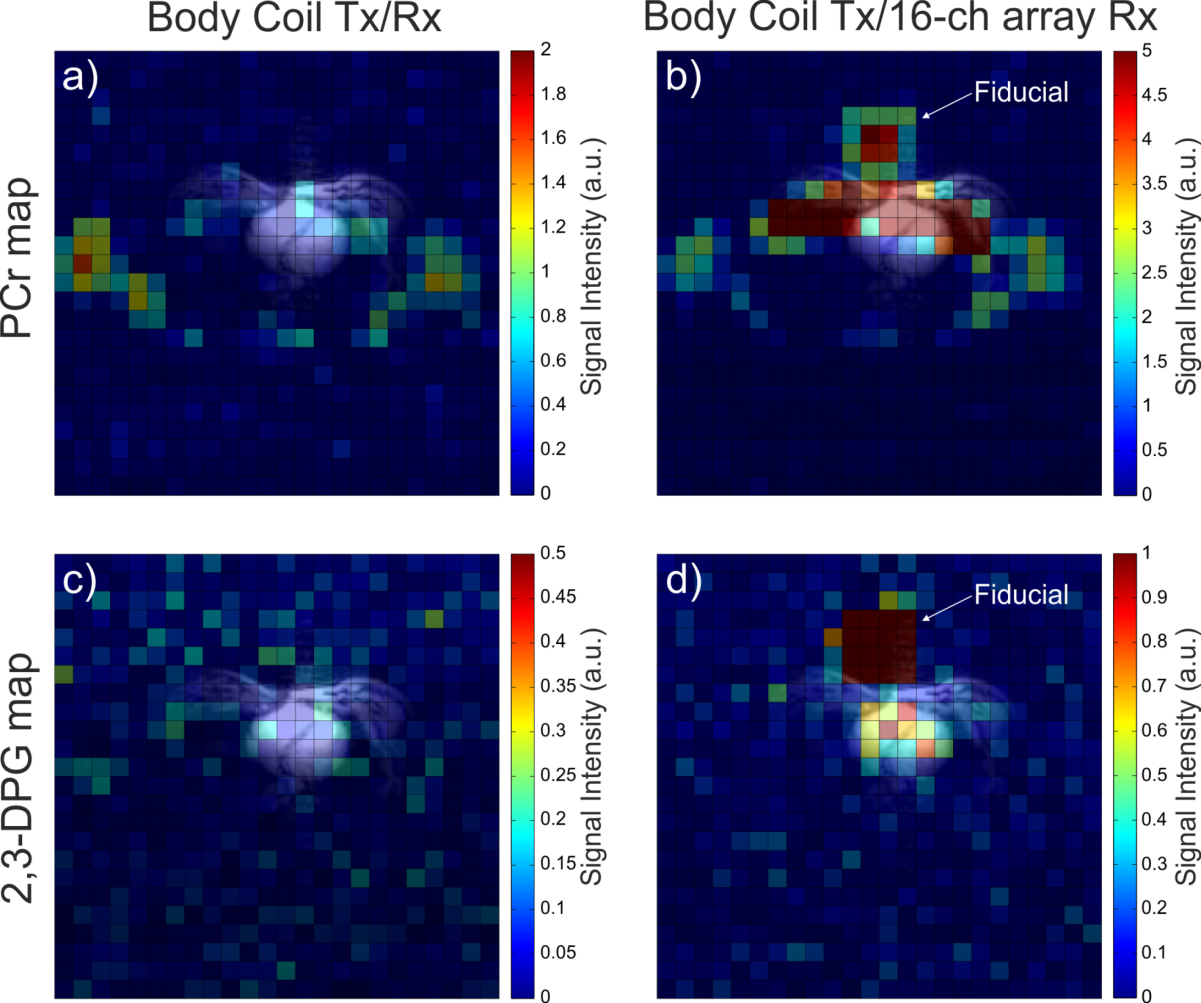
Metabolite maps. Transverse in vivo maps of (a, b) PCr and (c, d) 2,3-DPG signals acquired using the body coil in (a, c) transceiver mode and (b, d)using the combined whole-body transmit and 16-channel local receive setup. All maps are overlaid on ^1^H localizer images registered to the CSI grid. Note that the signals outside the body are from a concentrated fiducial mounted in the centre of the receive array.

Fig 2 depicts transverse 2D spatial distribution in vivo maps of PCr and 2,3-diphosphoglycerate (2,3-DPG) acquired using the body coil in transceiver mode and with the combined whole-body transmit and 16-channel receive setup. While the detection of signal from the heart region is challenging if the whole-body coil is used in transceiver mode, i.e. no 2,3-DPG signal visible, the heart is clearly delineated when the combined setup is used.

**Fig 3:**
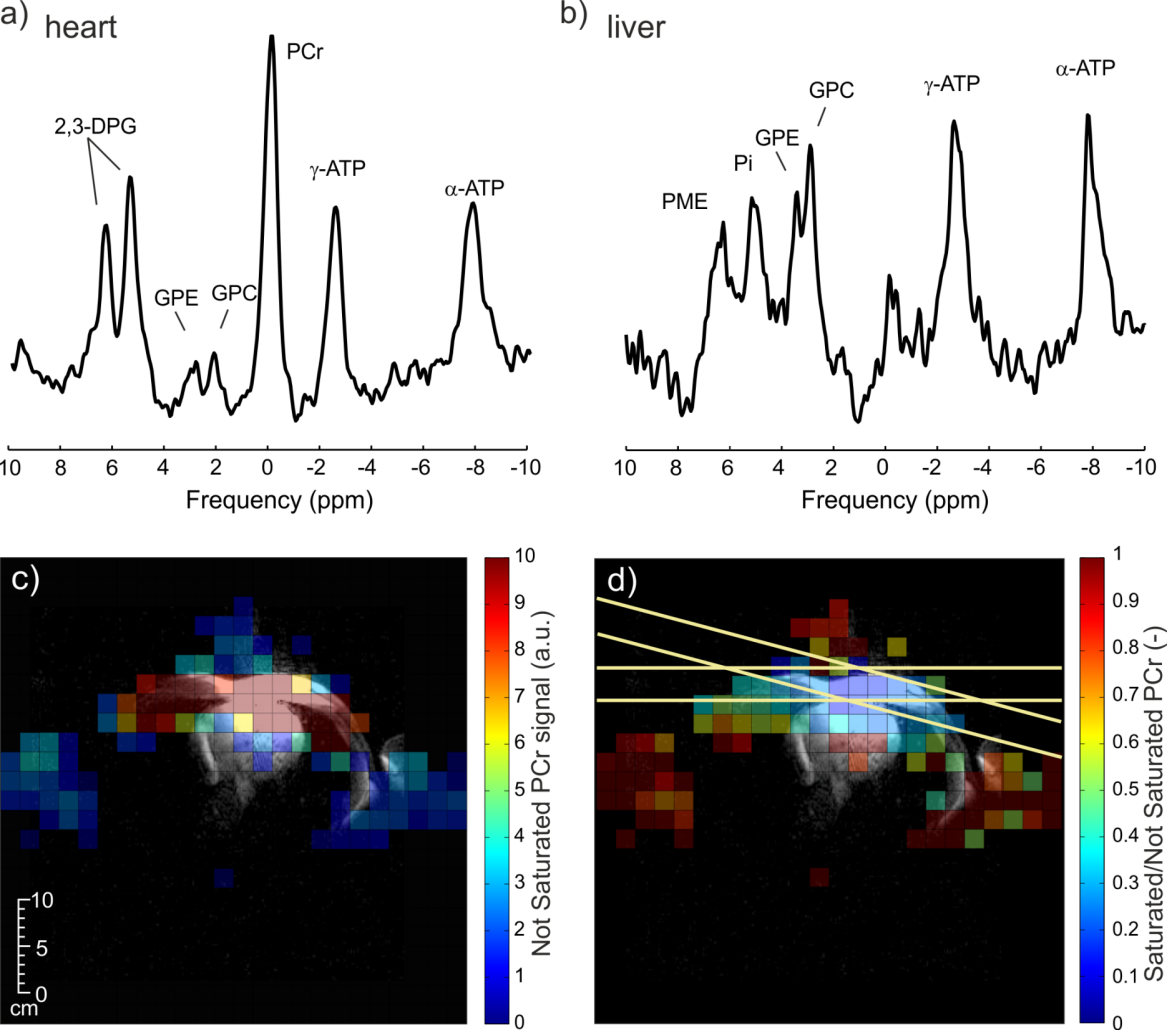
Acquired ^31^P-MR spectra. Representative in vivo ^31^P-MR spectra acquired with the 3D-UTE-CSI protocol in (a) human heart and (b) liver using the combined coil setup. (c) PCr metabolite map in transverse view through the chest without the saturation pulses. (d) Shows the ratio of PCr signal from acquisitions with and without the saturation bands applied to reduce skeletal muscle signal.

Representative in vivo spectra acquired with the 3D-UTE-CSI protocol in human heart and liver using the combined setup are depicted in Fig 3. The applied non-adiabatic saturation bands suppressed an average of 71 ± 4% of the PCr signal in the chest muscles (target region), while suppressing an average of 19 ± 3% of the PCr signal in the heart (not targeted); the suppression efficiency is depicted in Fig 3C and 3D.

**Fig 4:**
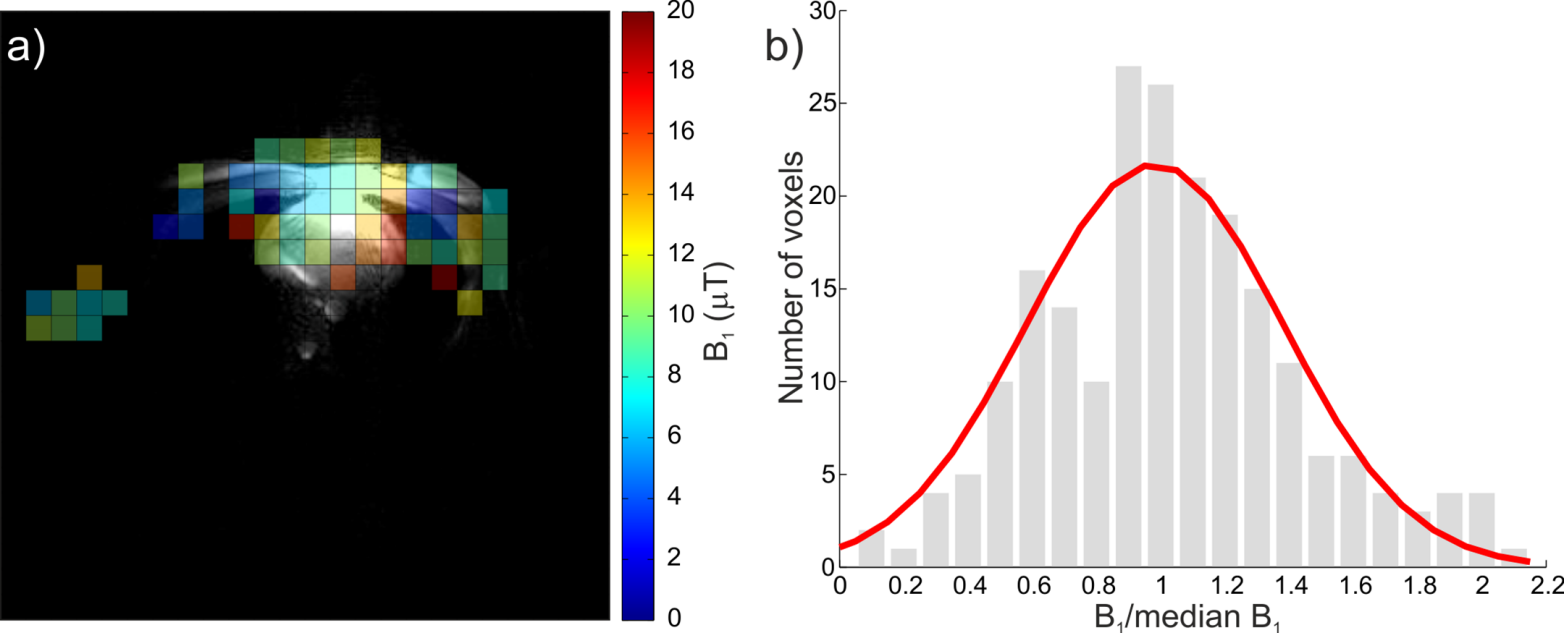
In vivo B_1_^+^ mapping. (a) Representative Bloch-Siegert, 2D, transverse B_1_^+^ map overlaid on ^1^H localizer image registered to the CSI grid. (b) A histogram of measured B_1_^+^ variability across all four subjects normalized to individual subject median B_1_^+^. A fitted normal distribution function (red line) is also depicted. All voxels with sufficient SNR were used for analysis.

The B_1_^+^ field was calculated in 52 ± 8 voxels satisfying the SNR criteria per subject. The mean measured CV for B_1_^+^ was 38.4%. The resulting mean measured ^31^P B_1_^+^ in vivo was 10.4 ± 2.7 μT. A representative in vivo B_1_^+^ map and a histogram of B_1_^+^ variability are shown in Fig 4.

## Discussion

In our study, we propose a new easily removable whole-body RF transmit coil combined with a 16-element receive array for cardiac ^31^P-MR at 7T. This setup provides homogeneous excitation through the whole chest without the need for adiabatic excitation pulses, as demonstrated by our CST simulations and ^31^P B_1_ maps measured in vivo. In addition, the receive array increases the SNR of our setup compared to the body coil in transmit/receive mode, allowing high-resolution ^31^P-MRSI experiments in vivo. The increased ^31^P B_1_^+^ homogeneity allows the use of conventional amplitude-modulated pulses to suppress chest wall muscles instead of the SAR demanding BISTRO approach [20] that has previously been used with surface transmit coils [8].

Our new coil was designed to easily move in and out of the bore of a Magnetom 7T MR scanner using the system’s motorized patient table. Therefore, the maximal inner diameter of the transmit coil was limited by the scanner bore diameter (58 cm). Our constructed whole-body coil had an inner diameter of 55 cm. Although this means reduction of the available patient space, it is still larger than the diameter of the previously reported whole-body coil that had a 52 cm diameter [15], and since it is integrated with the extension of the patient table, this allows us to scan a range of subjects in comfort. Additionally, the upper part of the birdcage coil is easily detachable, which facilitates straightforward positioning of subjects before their scan and rapid evacuation if a patient became acutely unwell. Using a volume transmit coil improved the transmit uniformity as expected, quantified by simulations and confirmed by our Bloch-Siegert ^31^P measurement in vivo. Our simulated SAR efficiency values were similar to the previously reported ones [15], i.e. 3.4 vs. 3.8 (local), 0.28 vs. 0.24 (global body) and 0.25 vs. 0.33 W/kg/μT^2^ (global head).

To increase receive sensitivity, we combined the whole-body transmit coil with a 16-element receive array. No changes in the S_11_ and S_12_ parameters of whole-body coil ports were observed using a network analyser (8712C, Hewlett Packard, Palo Alto, California) when the 16-channel array was inserted (regardless of its position). Adding the receive array led to a 2.6-fold increase in achieved SNR in phantom experiments, in comparison to the use of the whole-body coil in transceiver mode alone. This is comparable to the improvement seen on a 3T TIM Trio scanner (Siemens) between body coil ^1^H MRI and using half of a 32-channel cardiac receive array (Invivo, Gainesville, Florida). A further increase in SNR might be gained by placing another receive array beneath the volunteer. Active detuning of the body coil during signal acquisition could also potentially lead to an increase in SNR. Every increase in single-element SNR will also lead to an increase in the precision of the complex “weights” used in coil combination algorithms, e.g., WSVD. The final SNR will therefore increase both due to the increased single-element SNR, but even more because of the better complex “weights” estimation in the combination step [18].

On the other hand, the peak B_1_ achieved by the whole-body transmit coil in the phantom experiments at a depth of 10 cm was 3.5 times lower than that of our dedicated quadrature surface transmit coil as expected. However, the B_1_^+^ homogeneity of the whole-body transmit coil, allowing for short-TR scans with uniform Ernst-angle excitation or multi-echo readout [21] compensates for this. Furthermore, it may be feasible to drive the whole-body coil with a high-power ^31^P RF-amplifier, such as the 35 kW 123 MHz amplifiers used for ^1^H-MRI on Siemens 3T scanners. Dedicated low-loss transmit cabling could also be used. We estimate that these changes would give peak B_1_^+^ output comparable to our quadrature surface coil at 10 cm depth, while also retaining the coverage and uniformity advantages of the whole-body coil.

The in vivo data demonstrate that while our whole-body RF-coil in transceiver mode could be used to acquire PCr signal from skeletal muscles, its SNR is too low to allow detection of metabolites of lower concentration, e.g., 2,3-DPG. However, in combination with the 16-element receive array, good quality spectra and well-resolved maps of PCr and 2,3-DPG can be acquired in vivo. As expected, the PCr signal was localized to the skeletal muscle wall as well as the heart, while the 2,3-DPG signal was restricted only to the ventricular blood pools. This confirms the high spatial resolution of the acquired ^31^P-MRSI maps. The acquired in vivo ^31^P B_1_^+^ maps showed a high level of uniformity with a coefficient of variation <39% across all voxels with sufficient SNR. To demonstrate the use of this B_1_^+^ uniformity of the whole-body coil, we showed the effectiveness (>70%) of conventional amplitude-modulated saturation bands to suppress the signal from chest and abdominal muscles that can otherwise contaminate cardiac ^31^P-MRS data [8, 22].

In conclusion, we have designed, constructed and tested an easily removable, whole-body transmit RF-coil and its combination with 16-element receive array that is straightforward and comfortable to use for cardiac ^31^P-MR at 7T. This apparatus allowed us to measure in vivo the homogeneity of the ^31^P transmit field, confirming the results of electromagnetic field simulations. It allows us to record anatomically-consistent ^31^P metabolite maps, and to use saturation bands with amplitude-modulated pulses (and hence low SAR demands) to suppress signals from skeletal muscle. This combination of hardware is a step towards regionally-resolved, whole-heart cardiac ^31^P-MRS studies at 7T.
